# Organically Functionalized Magnesium Phyllosilicates: Surface Engineering and Antibacterial Performance

**DOI:** 10.1021/acsomega.5c02154

**Published:** 2025-07-17

**Authors:** Viktoria Sakavitsi, Renia Fotiadou, Mohammed Subrati, Kasibhatta Kumara Ramanatha Datta, Turki N. Baroud, Swarnamayee Behera, Konstantinos Spyrou, Mohamed A. Hammami, Panagiota Zygouri, Haralambos Stamatis, Ioannis V. Yentekakis, Dimitrios P. Gournis

**Affiliations:** † Department of Materials Science and Engineering, 37796 University of Ioannina, Ioannina 45110, Greece; ‡ Laboratory of Biotechnology, Department of Biological Applications and Technology, University of Ioannina, 45110 Ioannina, Greece; § Functional Nanomaterials Laboratory, Department of Chemistry, Faculty of Engineering and Technology, 93104 SRM Institute of Science and Technology, Kattankulathur, Tamil Nadu 603203, India; ∥ Interdisciplinary Research Center for Membranes and Water Security and Department of Materials Science and Engineering, King Fahd University of Petroleum & Minerals (KFUPM), Dhahran 31261, Saudi Arabia; ⊥ Department of Materials Science and Engineering, 5922 Cornell University, Ithaca, New York 14853, United States; # Laboratory of Physical Chemistry & Chemical Processes, School of Chemical and Environmental Engineering, 69002 Technical University of Crete (TUC), GR-73100 Chania, Crete, Greece; ¶ Institute of GeoEnergy, Foundation for Research and Technology-Hellas, GR-73100 Chania, Crete, Greece

## Abstract

Synthetic clay analogues (SCAs) of a new organosilicate layered material family, in contrast to common clays, are produced via an in situ room-temperature sol–gel route, providing the possibility for the design and synthesis of diverse, tailor-made functional groups on the surface and interior of the synthetic clay sheets. In this work, we introduce organophyllosilicates bearing different functional end groups, which are synthesized by a magnesium metal salt precursor and organosilanes such as (3-aminopropyl)­triethoxysilane (APTEOS), *N*-[3-(trimethoxysilyl)­propyl]­ethylenediamine (EDAPTEOS), *N*-(3-trimethoxysilylpropyl)­diethylenetriamine (TAPTMOS), 1,4-bis­(triethoxysilyl)­benzene (BTB), tetraethyl orthosilicate (TEOS), 3-glycidoxypropyltrimethoxysilane (GLYMO), and (3-chloropropyl)­trimethoxysilane (CPTMOS). The surface free energy for various organosynthetic clay analogues lies in the 29–252 mJ/m^2^ range. SCA’s antimicrobial activity was tested against both Gram-negative and Gram-positive bacteria to evaluate the effect of surface functionalization on the viability of these microorganisms. The amino-SCAs displayed higher antibacterial activity compared to epoxy-SCAs, presenting a dose-dependent effect and a structure-dependent motif. Furthermore, Gram-positive bacteria were more susceptible to SCA treatment than Gram-negative.

## Graphical abstract

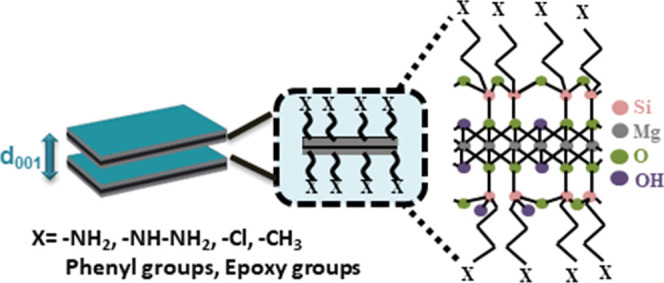

## 1 Introduction

Clay minerals, natural earth materials composed mainly of hydrous aluminum phyllosilicates, are ubiquitous on our planet in geologic deposits, in earthly biogeochemical cycles, and in the protective capacity of the oceans, as well as in the control of lethal discarded materials.^1^ They are also used as lubricants in petroleum extraction,^2^ fillers for the preparation of polymer-nanocomposites,^3^ and industrialized catalysts for the synthesis of various organic compounds.^4^ Due to their different compositions and poor dispersion ability in polar/nonpolar solvents, clay utilities in various areas are hampered.^5^ It is essential to tailor clay’s properties using appropriate organic functional groups that enable their dispersion in aqueous or organic solvents, thereby facilitating the design of synthetic clay analogues and novel hybrid materials for their processing.^5^ The traditional methods used for the organic modification of the clay include exchanging gallery cations with quaternary organic cations, including ammonium and phosphonium salts, etc., or direct modification of the clay layers using organic coupling agents, including silane coupling agents, and last, using crown ether to complex the clay cations. To increase the compatibility of clays with polymers, metal ions, and interfacial (aqueous–organic) reactions, it is necessary to tailor the organophilicity via surface functionalization with a specific functionality, which causes the variation of the surface energy of clay layers. The organic functional groups tethered over the clay surface interact with various matrixes at the interface via electrostatic and dispersive interactions. These alkyl chains, with different functional groups on the clay, provide the necessary interfacial adhesion with polymers, dispersion in polar solvents, and improved sorption behavior and selectivity, thereby altering the surface free energy, charge distribution, and strength.

Depending on functionalization, the obtained organically modified clays can be homogeneously dispersed in aqueous–organic solvents. Moreover, the organic moieties tethered over the clays reduce the clay’s surface energy, thereby improving the wetting characteristics and bringing amphiphilicity to the clays.^6^ The organic modification can be done either by ex situ or in situ approaches.^5,7^ In situ approaches are advantageous compared to ex situ methods, as the linkage of organic components via covalent bonds enables durable immobilization of the reactive organic groups, preventing their leaching into the surrounding medium when modified clay materials are used in solutions. The modification of the surface characteristics of silica or alumina/magnesium silicates is typically achieved by reacting silane derivatives, such as chlorosilanes, alkoxysilanes, or organosilanes, with silanol groups accessible on the surface.

Many examples of the in situ synthesis of synthetically modified clays via the sol–gel method have been reported. Mann et al. described different approaches for synthesizing layered, organized inorganic–organic nanocomposites based on self-assembled organic templates, 2:1 trioctahedral phyllosilicates, and organically functionalized magnesium phyllosilicate clays.^8–10^ Magnesium phyllosilicates bearing propylamine functionalities referred as aminoclays show excellent dispersibility in polar solvents and interesting properties rising from their layered structure,^5,11,12^ prepared by the sol–gel method using magnesium chloride and organotrialkoxysilane as precursors.^8^ According to the organic group of the organo-trialkoxysilane, different clays can be prepared by carrying various organic groups.^13^ Recently, Kaloudi et al. reported the use of a new lanthanum–cerium synthetic aminoclay analogue and studied the in vitro effects in normal and cancer cells, where the magnesium metal salt precursors have been replaced with rare earth ions.^14^

Due to their peculiar properties, synthetic clays have found a profound impact in applications such as sorbents for CO_2_ capture,^15^ antimicrobial agents,^16^ carriers in drug delivery systems hosting in their layer structure biomolecules, DNA, proteins, and enzymes.^12,17^ In addition to the above applications, aminoclays have also been successfully utilized in sensors and environmental applications.^18^

In the present work, via direct synthesis, based on the aforementioned facile, green, and effective sol–gel approach,^19^ a series of layered magnesium phyllo­(organo)­silicates comprising covalently tethered organic functionalities have been prepared. These functionalized organoclays with allyl and ethylenediamino pendants were characterized by using powder X-ray diffraction (XRD), Fourier transform infrared spectroscopy (FTIR), scanning electron microscopy (SEM), differential thermal and thermogravimetric analysis (DTA/TGA), and contact angle measurements. Furthermore, the surface free energies were evaluated, and the values for the synthetic organoclay analogues lie in the range of 29–252 mJ/m^2^. We further focus on a representative case study of great importance in biorelated applications, namely, the study of the antimicrobial activity of the pure SCA concentrate on the effect of the materials’ concentration, tailored with different functional groups, on the growth of *Escherichia coli* and *Corynebacterium glutamicum*, as model strains.

However, many works have studied the benefits of covalently tethered organic functionalities, mostly derived from silane precursors bearing amine groups leading to aminoclays with different numbers of organic chains on the surface and interlayer space of phyllosilicate clays; this work presents for the first time an extended study where we can successfully prepare magnesium phyllosilicates bearing various functional groups. By doing so, we aim to develop a family of synthetic organophyllosilicate materials with diverse physical and chemical properties that can be utilized in various applications according to their specific structure.

## 2 Materials and Methods

### 2.1 Materials Preparation

Magnesium chloride hexahydrate (MgCl_2_·6H_2_O) was purchased from Riedel-de Han; (3-aminopropyl)­triethoxysilane (APTEOS, 99%), *N*-[3-(trimethoxysilyl)­propyl]­ethylenediamine (EDAPTEOS, 97%), *N*-(3-trimethoxysilylpropyl)­diethylenetriamine (TAPTMOS, technical grade), 1,4-bis­(triethoxysilyl)­benzene (BTB, 96%), and tetraethyl orthosilicate (TEOS, 98%) from Sigma-Aldrich; 3-glycidoxypropyltrimethoxysilane (GLYMO, 97%) and (3-chloropropyl)­trimethoxysilane (CPTMOS, 98+%) from Acros Organics; and sodium hydroxide (NaOH) and ethanol from Merck. All chemicals were used as received without any further purification and are presented in Figure 1.

**Figure 1:**
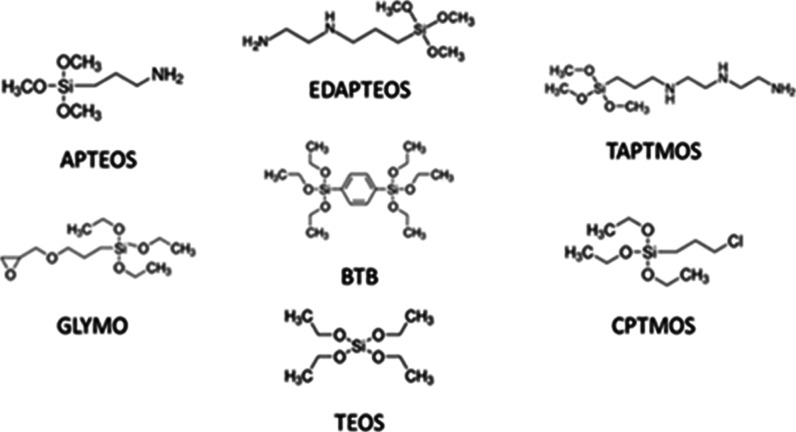
Chemical structures of the silane precursors [(3-aminopropyl)­triethoxysilane (APTEOS), *N*-[3-(trimethoxysilyl)­propyl]-ethylenediamine (EDAPTEOS), *N*-(3-trimethoxysilylpropyl)­diethylenetriamine (TAPTMOS), 3-glycidoxypropyltrimethoxysilane (GLYMO), 1,4-bis­(triethoxysilyl)­benzene (BTB), (3-chloropropyl)­trimethoxysilane (CPTMOS), and tetraethyl orthosilicate (TEOS)], respectively, toward the synthesis of SCAs.

#### 2.1.1 Preparation of Amino Synthetic Clay Analogues (Am-SCAs)

Three Am-SCAs were synthesized using amino silanes of varying numbers of amine functional groups, namely, (i) APTEOS (monoamine), (ii) EDAPTEOS (diamine), and (iii) TAPTMOS (triamine). In a typical synthesis, three solutions, APTEOS (2.6 mL, 10 mmol), EDAPTEOS (2.16 mL, 10 mmol), and TAPTMOS (2.58 mL, 10 mmol), were added dropwise, respectively, to a solution of ethanol (50 mL) and MgCl_2_·6H_2_O (1.68 g, 9.15 mmol) at room temperature yielding the sol phase of the clay colloid. Using an aqueous solution of NaOH (1 M), the pH of the sol was adjusted to basic conditions (pH > 7) to induce the sol–gel transition. It is worth noting that the gelation point is strongly influenced by the type of silane precursor used. In this respect, the gelation points corresponding to the silanes mentioned above are as follows: (i) APTEOS (pH ∼9.8), (ii) EDAPTEOS (pH ∼10.4), and (iii) TAPTMOS (pH ∼10.3). The final dispersion was stirred at room temperature until a white slurry was obtained. The white gel was then recovered by centrifugation, washed with ethanol, and dried overnight at 40 °C, yielding poorly ordered gelled aggregates of clay sheets. To improve their ordering, the hydrophilic clay sheets were first exfoliated in deionized water, followed by an equal volume of ethanol to facilitate their ordering and precipitation. Finally, the dispersion was centrifuged, and the precipitated ordered sheets were dried overnight at 40 °C. Each as-synthesized sample was denoted as Am­(*x*)-SCA, where *x* is the number of amino groups per molecule of the amino silane precursor. Through the protonation of amine groups, phyllosilicates undergo free and reversible exfoliation in water. However, they can freely return to their lamellar structure by adding less polar solvents, such as ethanol. In this way, the ordering of the amine-functionalized phyllosilicates can be controlled.

#### 2.1.2 Preparation of Epoxy, Chloro-, and Aliphatic Synthetic Clay Analogues (Ep-SCA, Ch-SCA, and Al-SCA)

The (i) Ep-SCA, (ii) Ch-SCA, and (iii) Al-SCA samples were prepared using (i) GLYMO (2.21 mL, 10 mmol), (ii) CPTMOS (1.86 mL, 10 mmol), and (iii) TEOS (2.23 mL, 10 mmol) as silane precursors, respectively, and following the same synthesis procedure used for Am-SCAs. Furthermore, the pH adjustment of the corresponding sols was carried out in accordance with the silanes used as follows: (i) GLYMO (pH ∼9.9), (ii) CPTMOS (pH ∼8.6), and (iii) TEOS (pH ∼7.7), using an aqueous solution of NaOH (1 M). When the slurry (gel) was formed, the dispersion was centrifuged, and the precipitated ordered sheets were dried overnight at 40 °C.

#### 2.1.3 Preparation of the Synthetic Aromatic Clay Analogue (Ar-SCA)

The Ar-SCA sample was prepared by using BTB as an aromatic silane precursor. Briefly, BTB (3.98 mL, 10 mmol) was added dropwise to 50 mL of an ethanolic solution of MgCl_2_·6H_2_O (1.68 g, 9.15 mmol) at room temperature. Subsequently, gelation of the resulting sol was induced by adjusting the pH to basic conditions (pH ∼9.5) using an aqueous solution of NaOH (1 M). The postgelation procedure is similar to that of Am-SCAs. Finally, the dispersion was centrifuged, and the precipitated ordered sheets were dried overnight at 40 °C. The schematic experimental procedure is presented in Figure 2.

**Figure 2:**
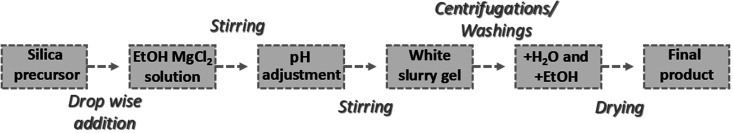
Flowchart showing the synthetic organosilicate clay analogue.

### 2.2 Materials Characterization

#### 2.2.1 Powder X-ray Diffraction (XRD)

The diffraction patterns were collected at room temperature on a D8 advance Bruker diffractometer with a monochromatic Cu Kα source (wavelength: 1.54 Å); a 1 mm divergent slit and a 3 mm antiscattering slit were used. The 2θ scans were performed from 2 to 80° with a step size of 0.02° and a counting time of 1.00 s per step.

#### 2.2.2 Scanning Electron Microscopy (SEM)

SEM images were acquired using a JEOL JSM-5600 microscope (JEOL Ltd., Tokyo, Japan) with 10 and 25 kV accelerating voltage.

#### 2.2.3 FTIR Spectroscopy

Infrared spectra were measured with a Jasco FT/IR 6200 infrared spectrometer equipped with a deuterated triglycine sulfate (DTGS) detector, in the region of 400–4000 cm^–1^. Each spectrum had an average of 32 scans, and the resolution was 2 cm^–1^. KBr pellets containing ca. 2 wt % samples were prepared for these measurements.

#### 2.2.4 Thermal Analysis

Differential thermal analysis and thermogravimetric analysis (DTA/TGA) were performed with a PerkinElmer Pyris-Diamond. Samples of approximately 5 mg were heated in air from 25 to 850 °C, at a rate of 5 °C min^–1^.

#### 2.2.5 Contact Angle Measurements

Contact angle measurements on the powder samples were performed using the sessile drop method with a KYOWA DMs-401 contact angle goniometer at 25 °C, utilizing FAMAS Add-in software. The surface free energy was calculated by employing the Kitazaki–Hata theory wherein the solvent contact angles of water, ethylene glycol, and hexadecane were measured respectively. For this measurement, the powders were gently compressed between two clean glass slides to form a compact, uniform, and flat surface suitable for static contact angle analysis. Surface free energy calculations were performed using the extended Fowkes model (Kitazaki–Hata theory).^20^ For this, contact angle measurements were carried out using three liquids with differing polarities and surface tensions: water (∼72.8 mN/m), ethylene glycol (∼47.7 mN/m), and hexadecane (∼27.6 mN/m). Subsequently, surface free energy (SFE) was calculated based on the extended Fowkes model, which postulates that the total surface energy (γ^total^) of a material is the summation of its dispersive (γ^d^), polar (γ^p^), and hydrogen bonding (γ^h^) components, as expressed by the following equation:

$$\gamma^{total}=\gamma^{d}+\gamma^{p}+\gamma^{h}$$

The individual components of the surface energy were then derived by applying the Kitazaki–Hata equation to the measured contact angles.

### 2.3 Antibacterial Activity Evaluation

#### 2.3.1 Materials

*Escherichia coli* (*E. coli*) strain BL21­(DE3) and *Corynebacterium glutamicum* (*C. glutamicum*) strain ATCC 21253 were taken from cultural collections of the Department of Biological Applications and Technologies (Ioannina, Greece). The strains were recovered from cryopreservation (20% glycerol stocks) and regrown in Luria–Bertani (LB) medium, Lennox formulation (NEOGEN Co. 620 Lesher Place, Lansing, MI 48912 USA) at 37 ± 1 °C under orbital shaking at 160 rpm (KS 4000 ic control, IKA, Königswinter, Germany).

#### 2.3.2 Evaluation Method

The antibacterial activity of amino and epoxy synthetic clay analogues (Am-SCAs and Ep-SCAs) was tested against a Gram-negative and a Gram-positive bacterium, *E. coli* BL21 and *C. glutamicum* ATCC 21253, respectively.^21^ Fresh bacteria cells from the exponential phase (∼10^8^ CFU mL^–1^) were added to aqueous (0.9% w/v, NaCl) dispersions of synthetic clay analogue samples of different material concentrations. The samples were continuously agitated in an orbital shaker at 160 rpm and a constant temperature of 37 ± 1 °C for a period of 16 h (KS 4000 ic control, IKA, Königswinter, Germany). A control sample for each bacterium was also prepared. After that incubation period, 25 μL of each sample was transferred to a 96-well sterile microplate containing 225 μL of fresh LB broth medium (bacteria cells ∼ 10^7^ CFU mL^–1^) and incubated at 37 ± 1 °C for eight hours under shaking. The absorbance was recorded at 600 nm per hour using a UV/vis microplate reader (Multiskan SkyHigh, Thermo Fisher Scientific, Cleveland, OH, USA). The antibacterial efficiency of each material was defined as the percentage of the growth inhibition of the treated cells compared to the control sample at the exponential growth phase (4 h). The lethal concentration (LC_50_) represents the concentration of the sample required to reduce the growth of the bacterial population by approximately 50%. All measurements were performed in triplicate.

#### 2.3.3 Statistical Analysis

All analyses were carried out in triplicate, and the results were recorded as mean ± standard deviation. One-way ANOVA analysis and Tukey’s multiple comparison test were carried out using IBM SPSS Statistics version 21 (SPSS Inc., Chicago, IL, USA) to compare the mean values of each treatment and to determine the statistical significance where appropriate (*p* < 0.05).

## 3 Results and Discussion

XRD examined the structure and morphology of the as-synthesized SCAs. The X-ray diffractograms of all the samples (Figure 3) reveal low-angle reflections (2θ < 10°) corresponding to the d_001_ interlayer spacings of bilayer assemblies of the silane molecules that constitute the SCAs.^15^ More specifically, the diffractograms of Am(1)-SCA, Am(2)-SCA, and Am(3)-SCA show an increase of the basal spacing (d_001_), which is 15.2 Å for Am(1)-SCA, becomes 16.6 Å for Am(2)-SCA and in the same increasing fashion goes until 19.6 Å for Am(3)-SCA, therefore with the increasing number of amino groups, d_001_ increases. The basal diffraction peaks and their corresponding spacings for all samples are listed in Table 1. The high-angle in-plane reflections at 2θ = 15–31°, 32–40°, and 60° correspond to the d_020,110_, d_130,200_, and d_060_ interlayer spacing, respectively.^22^ This confirms the formation of the 2:1 trioctahedral Mg-organophyllosilicate clay with a layer-like structure, which also agrees with the SEM images (Supporting Information, as shown in Figures S1–S7). The reflection at 2θ = 60°, which is more pronounced in the first four samples (Am-SCAs and Ep-SCA), indicates a more organized structure in contrast to the rest of the last samples (Ar-SCA, Ch-SCA, and Al-SCA).

**Figure 3:**
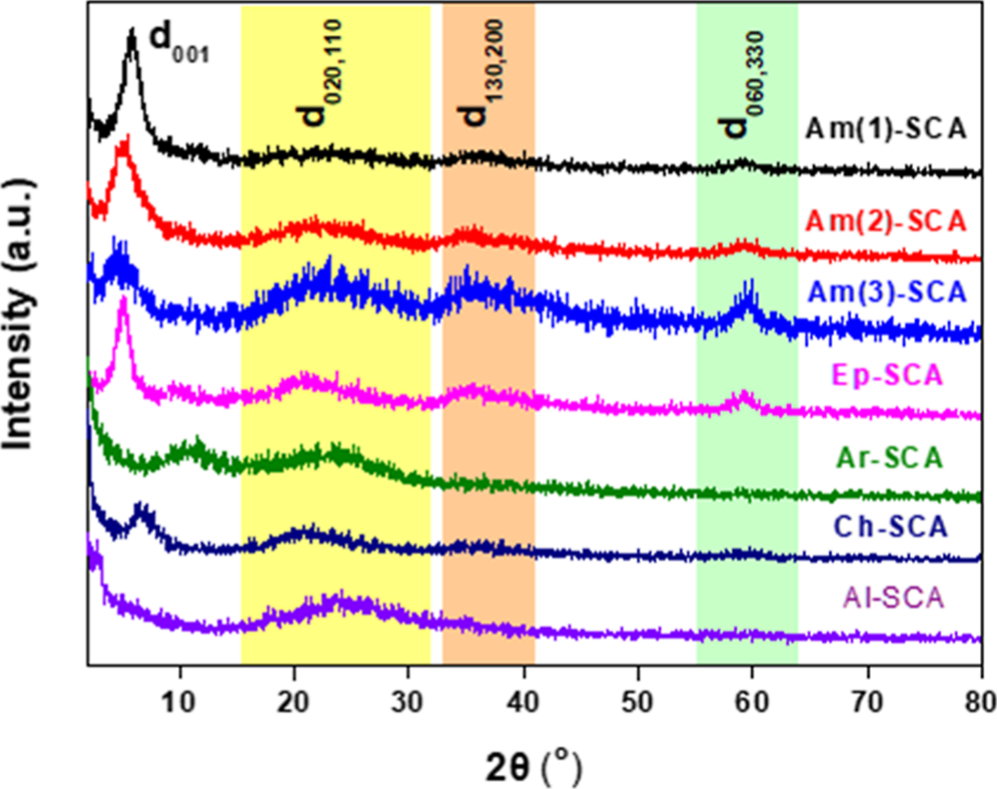
X-ray diffractograms of organosynthetic clay analogues bearing different silane precursors.

**Table 1:** XRD Analysis of Synthetic Clay Analogues with Different Silane Precursors

sample	2θ (deg)	*d*_001_ (Å)
Am(1)-SCA	5.8	15.2
Am(2)-SCA	5.3	16.6
Am(3)-SCA	4.5	19.6
Ep-SCA	5.1	17.4
Ar-SCA	10.5	8.4
Ch-SCA	6.5	13.6
Al-SCA	2.2	40.6

The FTIR spectra of the as-synthesized SCAs (Figure 4) exhibit absorption bands at 450–550 cm^–1^ and 1000–1186 cm^–1^ corresponding to Mg–O and Si–O–Si stretching vibrations.^9,23^ This confirms the co-condensation of magnesium cations and organofunctional alkoxysilane molecules into magnesium phyllosilicates.^24^ The organofunctional nature of the developed SCAs is as follows: For the Am-SCAs samples, the absorption bands at 1550–1650 cm^–1^ can be ascribed to the bending vibrations of the amino groups.^22^ The FTIR spectrum of the Ep-SCA shows bands at 1344, 1650, and 1792 cm^–1^, which are attributed to the epoxy ring stretching of the C–H, C–C, and C–O, respectively.^25^ In the spectrum of Ar-SCA, the two absorption bands at 1635 and 3061 cm^–1^ correspond to the stretching vibrations of the C=C and C–H bonds in benzene rings.^26^ For the Ch-SCA sample, the vibration bond at 696 cm^–1^ can be assigned to the C–Cl bond.^27,28^ Finally, Figure S2 illustrates the presence of functional groups and thermal stability for all synthetic clay analogues.

**Figure 4:**
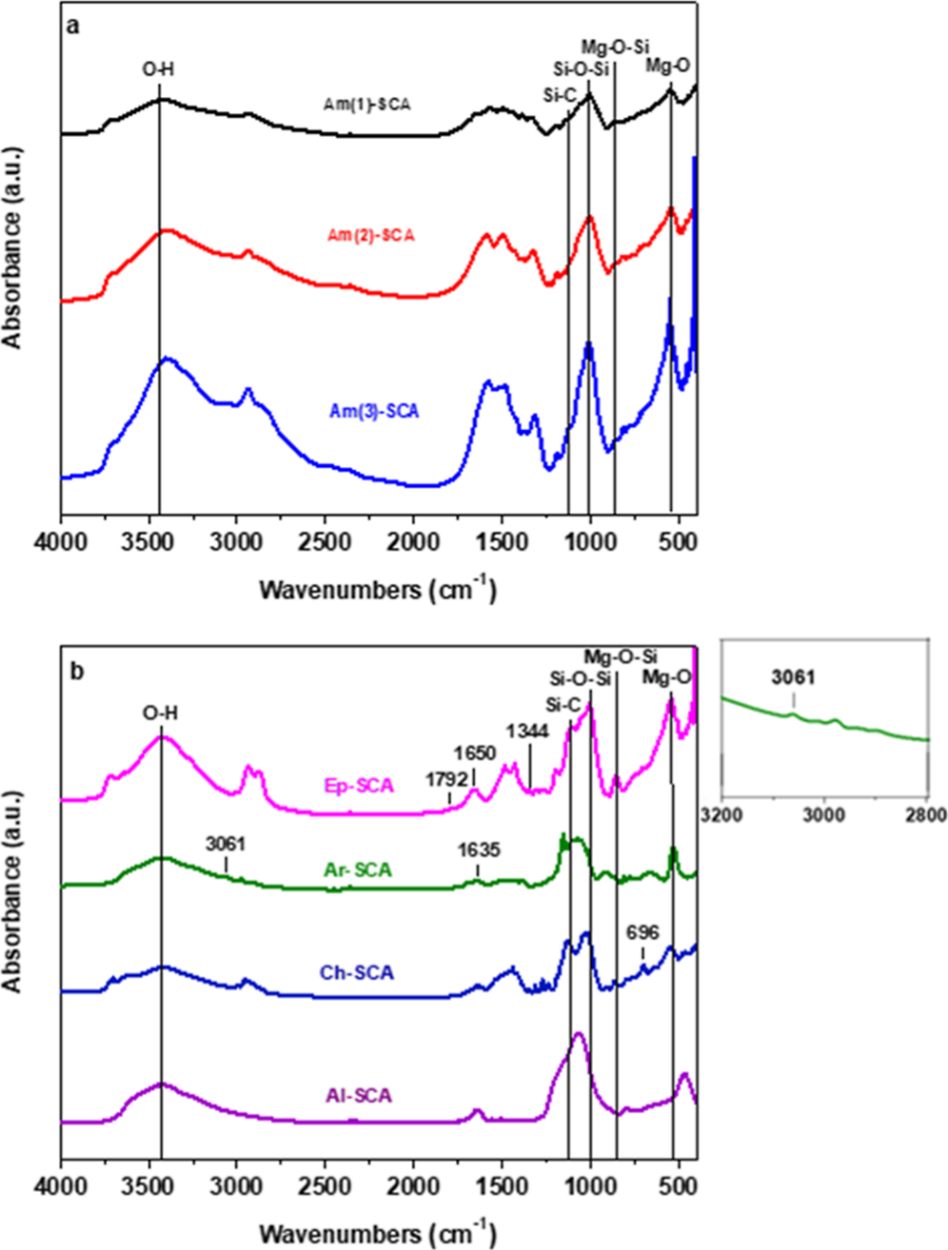
FTIR spectra of (a) Am(1)-SCA, Am(2)-SCA, and Am(3)-SCA and (b) Ep-SCA, Ar-SCA, Ch-SCA, and Al-SCA (inset: Figure 1b, Ar-SCA).

The solvent contact angles and surface free energy (SFE) analysis were performed for the synthetic organoclay analogues, and the results are presented in Table 2. SFE, also termed solid surface tension, is the excess energy stemming from the material’s surface compared to its bulk counterpart. SFE influences the material’s ability to interact with liquids with diverse polarity and is usually determined by measuring the contact angles of different solvents on the material’s surface.^29^ Particularly, if the water contact angle (WCA) is less than 90°, the material is said to be hydrophilic, whereas the WCA that exceeds 90° is referred to as hydrophobic. Hydrophilicity–hydrophobicity, along with the contact angle (CA) measurements on organic solvents, directly impact surface free energy. For instance, multiple solvents with different polarities and surface tension, such as water, ethylene glycol, and hexadecane, were chosen to determine the SFE of the organoclay derivatives.^30^ The surface tension values of polar water, moderately polar ethylene glycol, and hexadecane are nearly 72.8, 47.7, and 27.6 mN/m, respectively. The selected solvents’ polarity gradient, when dispensed on the material surface, and the wettability (analyzed via contact angle values) give information about the polar, hydrogen bonding, and dispersive interactions on the organoclay surfaces.

**Table 2:**
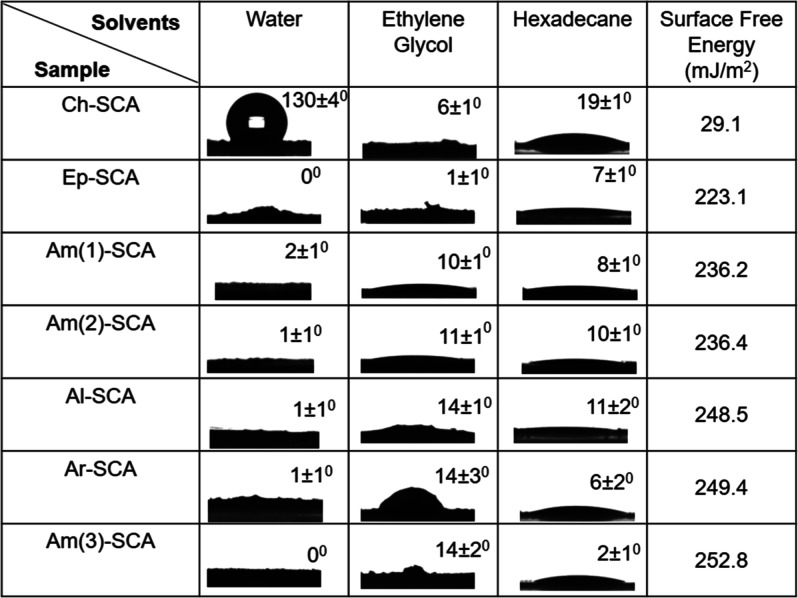
Solvent Contact Angles and Surface Free Energy of Synthetic Clay Analogues with Different Silane Precursors^a^

a The measurement was reproduced on two different locations of the sample, and the error bar represents the standard deviation.

The SFE analysis was performed for the synthetic organoclay analogues, and the results are presented in Table 2. Specifically, the solvent contact angles with polarity gradient, including water, ethylene glycol, and hexadecane for various clay derivatives, were measured to determine the SFE.^30,31^ For various clay derivatives, the SFE values lie in the range of 223 to 252 mJ/m^2^; however, for the Ch-SCA sample, we observe an SFE value of 29 mJ/m^2^. The higher SFE values indicate the amphiphilic nature of various clay derivatives, where the solvents interact well with the silica and alkyl/aryl pendants of clay. It is essential to highlight that most of the clay derivatives (Ep-SCA, Am(1)-SCA, Am(2)-SCA, Am(3)-SCA, Al-SCA, and Ar-SCA) exhibit a hydrophilic nature, except for Ch-SCA, which shows hydrophobicity. It is important to mention that we observe a higher CA of water and hexadecane for Ch-SCA as compared to the rest of the clay analogues, which in turn impacts the enhanced water-wetting resistance (hydrophobicity). The orientation of the alkyl/aryl pendant groups of silanes and their lamellar organization play a significant role in solvent wettability and surface free energy. Nevertheless, SFE on clays is an important analysis for understanding the surface properties of clay materials, which in turn have important applications in coatings and durable polymer-nanocomposite films.^32^

The antimicrobial activity of organic analogues of Mg-phyllosilicate clays has been reported, focusing on the behavior of bacterial and fungal strains.^16,24^ Among the various synthetic organoclays analogues, the ones bearing amino and epoxy groups appear to be appealing for antibacterial applications, exploiting in such a way the layered structure and shape of the synthetic clay, as well the specific ligands that exist on the surface and interlayer space of the phyllomorphous materials. The present study evaluated the antibacterial activity of various synthetic amino and epoxy clay analogues against a Gram-positive and a Gram-negative model strain after 16 h of interaction. Figure 5 illustrates the lethal effects of the different materials against *E. coli*. The antibacterial activity follows a dose-dependent pattern, exhibiting almost 100% growth inhibition at doses over 75 μg mL^–1^. Table 3 gives a more thorough insight into the antimicrobial efficiency of the modified Mg-phyllosilicate clays. The growth inhibition on the tested cell populations expressed as LC_50_ values demonstrated that the Ep-SCA provokes a decrease of the *E. coli* cells at higher doses compared to the Am-SCA. Moreover, the number of the amine functional groups (Am(1)-SCA, Am(2)-SCA, and Am(3)-SCA) led to a significant effect regarding the antibacterial activity among the three different Am-SCA samples. In the case of *C. glutamicum*, the antimicrobial activity of SCAs is also dose-dependent, with lethal concentrations up to 50 or 75 μg mL^–1^, depending on the material (Figure 5). Furthermore, amino analogues provoked suppression of 50% of the treated bacterial populations at lower doses compared to the epoxy clay analogue apart from the Am(3)-SCA exhibiting a clear motif, as described above.

**Figure 5:**
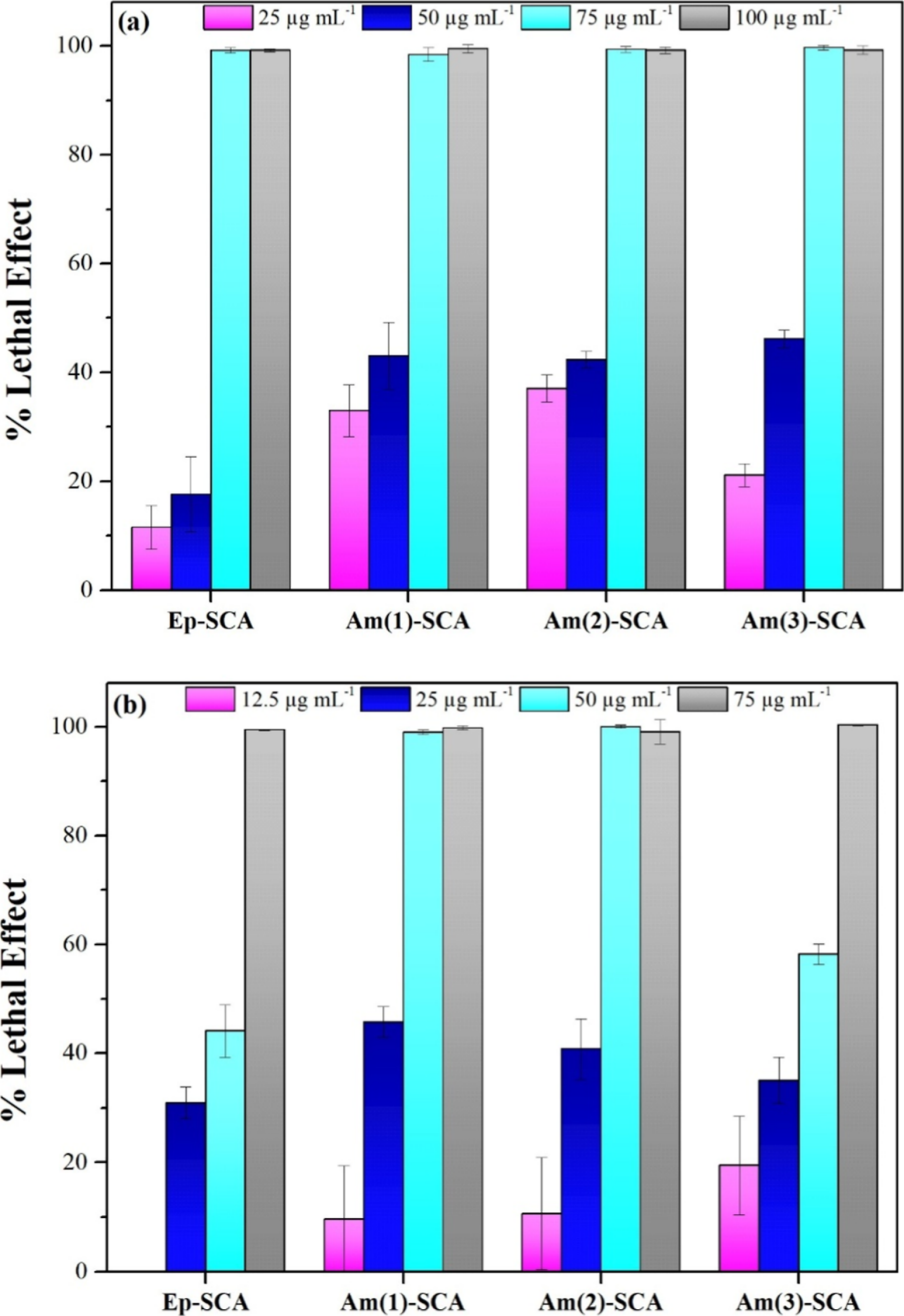
Lethal effect of Ep-SCA, Am(1)-SCA, Am(2)-SCA, and Am(3)-SCA on (a) *E. coli* and (b) *C. glutamicum* cells under different concentrations and after 16 h of interaction. All analyses were carried out in triplicate, presenting the results as mean ± standard deviation.

**Table 3:** LC_50_ of SCAs against *E. coli* and *C. glutamicum* ^a^

	LC_50_ (μg mL^–1^)	
synthetic clay analogues	*E. coli*	*C. glutamicum*
Ep-SCA	53.8 ± 0.6^d^	42.2 ± 0.5^c^
Am(1)-SCA	42.6 ± 0.8^a^	25.6 ± 0.3^a^
Am(2)-SCA	44.5 ± 0.3^b^	26.4 ± 0.4^a^
Am(3)-SCA	46.7 ± 0.4^c^	40.2 ± 0.3^b^

a Means with different lowercase letters are significantly different (*p* < 0.05) according to Tukey’s test.

It is evident that the LC_50_ values of the SCAs are lower for the Gram-positive bacterium cells causing growth inhibition at doses below 42.5 μg mL^–1^. This is due to the differences in the cell wall of the Gram-positive bacteria compared to the Gram-negative bacteria, which makes the former less resistant to possible bactericidal agents, allowing their faster and easier interaction with the bacterium surface.^33^

Moreover, as comprehensively elucidated before, amine groups of Am-SCAs can interact with bacterial cells, disrupting the bacterial cell wall/membrane and causing cell death.^16^ As far as the epoxy groups of the Ep-SCA are concerned, it has been previously explained that the epoxy groups generate oxidative stress, which leads to irreversible cell damage.^34^

To further ascertain the bactericidal activity, Am(1)-SCA–Am(3)-SCA were deposited on the surface of Si-wafers using the Langmuir–Blodgett (LB) technique presented in Supporting Information. For this purpose, an antibacterial drop-test was carried out using *E. coli* cells as a case of study. The percentage of cell viability was determined by measuring the number of remaining viable cells after overnight incubation with the Si-wafer-modified surfaces. Table S1 summarizes *E. coli* viability after interaction with the different samples. Blank Si-wafers were also tested as a reference, and an insignificant effect on the viability of the cells was detected. As shown above, Am-SCA materials exhibited a significant decrease in the bacterial population, which exceeded 90%. The three aminoclay analogues did not present substantial differences in their bactericidal activity when deposited on the surface of Si-wafers, hypothesizing that the –NH_2_ terminal group possesses the pivotal role, as known before, but further experiments are required to access the optimal SCA content on the surface of Si-wafers and the effective contact time to reduce the bacterial population. To conclude, it is evident that these materials exhibit strong antibacterial properties, paving the way for potential coating applications. The significance of the LB method is that it allows for the controlled deposition of ultrathin SCA layers on a substrate of a specific size, which affects both the antibacterial properties of the material (by controlling the size of the layers and uniformity of the films) and the cost of the application, where we need a tiny amount of the nanomaterial to fulfill big substrates.

## 4 Conclusions

In this work, we have successfully synthesized seven different types of phyllosilicate layered materials bearing different organic functionalities on the surface and interlayer spacing of the final synthetic clays (SCA). The SCA was characterized via various surface and microscopic techniques (XRD, FTIR, SEM, DTA/TGA, and contact angle measurements), revealing the layered structure and the type of functional end groups. The surface free energy for various organo synthetic clay analogues lies in the range of 29–252 mJ/m^2^. These functional layered structural hybrids were tested as potential antimicrobial agents. The SCAs presented dose-dependent inhibition of the growth of *E. coli* and *C. glutamicum*. The amino-functionalized materials exhibited the most pronounced antibacterial activity due to the presence of amine groups. Future research should focus on the deposition of SCAs on surfaces of interest and test their antibacterial activity under real-case conditions.

This work focuses on the development of a new class of layered synthetic organophyllosilicate clays with precisely tunable surface functionalities designed for a wide range of applications in biology, environmental remediation, and innovative nanocomposites. Their intrinsic layered architecture enables further modification, harnessing the well-known 2D chemistry of clay minerals.

## Supplementary Material



## Notes

Some additional data including scanning electron microscopy images, differential thermal analysis and thermogravimetric analysis (DTA/TGA), and cell viability test (PDF)

## ∇

Institute of Nanoscience and Nanotechnology, NCSR “Demokritos”, Aghia Paraskevi, 15310 Attikis, Greece

## ○

Department of Chemistry, University of Crete, GR-70013, Heraklion, Crete, Greece.

## Abbreviations

SCAs: synthetic clay analogues
Am-SCAs: amino synthetic clay analogues
Ep-SCAs: epoxy synthetic clay analogues
Ch-SCA: chloro-synthetic clay analogue
Al-SCA: aliphatic synthetic clay analogue
APTEOS: (3-aminopropyl)­triethoxysilane
EDAPTEOS: *N*-[3-(trimethoxysilyl)­propyl]­ethylenediamine
TAPTMOS: *N*-(3-trimethoxysilylpropyl)­diethylenetriamine
BTB: 1,4-bis­(triethoxysilyl)­benzene
TEOS: tetraethyl orthosilicate
GLYMO: 3-glycidoxypropyltrimethoxysilane
CPTMOS: (3-chloropropyl)­trimethoxysilane
XRD: X-ray diffraction
FTIR: Fourier transform infrared spectroscopy
SEM: scanning electron microscopy
DTA: differential thermal analysis
TGA: thermogravimetric analysis
MgCl_2_·6H_2_O: magnesium chloride hexahydrate
NaOH: sodium hydroxide
DTGS: deuterated triglycine sulfate
*E. coli*: *Escherichia coli*
*C. glutamicum*: *Corynebacterium glutamicum*
LC_50_: lethal concentration
