# Supplementary material for: Organically Functionalized Magnesium Phyllosilicates: Surface Engineering and Antibacterial Performance
Source: ACS Omega. 2025 Jul 17;10(29):31568–76. doi: 10.1021/acsomega.5c02154 (PMC12338954; doi:10.1021/acsomega.5c02154)
Supplement: Supplementary file 1 [file ao5c02154_si_001.pdf]

Supporting Information

Organically Functionalized Magnesium Phyllosilicates: Surface Engineering and Antibacterial Performance

Viktoria Sakavitsi[a], Renia Fotiadou[b], Mohammed Subrati[a], Kasibhatta Kumara Ramanatha Datta[c], Turki N. Baroud[d], Swarnamayee Behera[c], Konstantinos Spyrou[a], Mohamed A. Hammami[e], Panagiota Zygouri[a], Haralambos Stamatis[b], Ioannis V. Yentekakis[f,g] and Dimitrios P. Gournis[a,,f,g*]*

[a] *Department of Materials Science and Engineering, University of Ioannina, Ioannina 45110, Greece*

[b] *Laboratory of Biotechnology, Department of Biological Applications and Technology, University of Ioannina, 45110 Ioannina, Greece*

[c] *Functional Nanomaterials Laboratory, Department of Chemistry, Faculty of Engineering and Technology, SRM Institute of Science and Technology, Kattankulathur, Tamil Nadu 603203, India*

[d] *Interdisciplinary Research Center for Membranes and Water Security and Department of Materials Science and Engineering, King Fahd University of Petroleum & Minerals (KFUPM), Dhahran 31261, Saudi Arabia*

[e] *Department of Materials Science and Engineering, Cornell University, Ithaca, NY 14853, USA.*

[f] *Laboratory of Physical Chemistry & Chemical Processes, School of Chemical and Environmental Engineering, Technical University of Crete (TUC), GR-73100, Chania, Crete, Greece*

[g] *Institute of GeoEnergy, Foundation for Research and Technology-Hellas, GR-73100 Chania, Crete, Greece*

1. SEM measurements

Representative SEM images for all the synthetic clays reveal the sheet-like structure of Am(1)-SCA (Figure S1)., Am(2)-SCA (Figure S2),. Am(3)-SCA (Figure S3)., Ep-SCA (Figure S4)., Ar-SCA (Figure S5)., Ch-SCA (Figure S6) and Al-SCA (Figure S7). In all images it can be observed a layered-like structure of the Mg-organophyllosilicate clays (arrows).

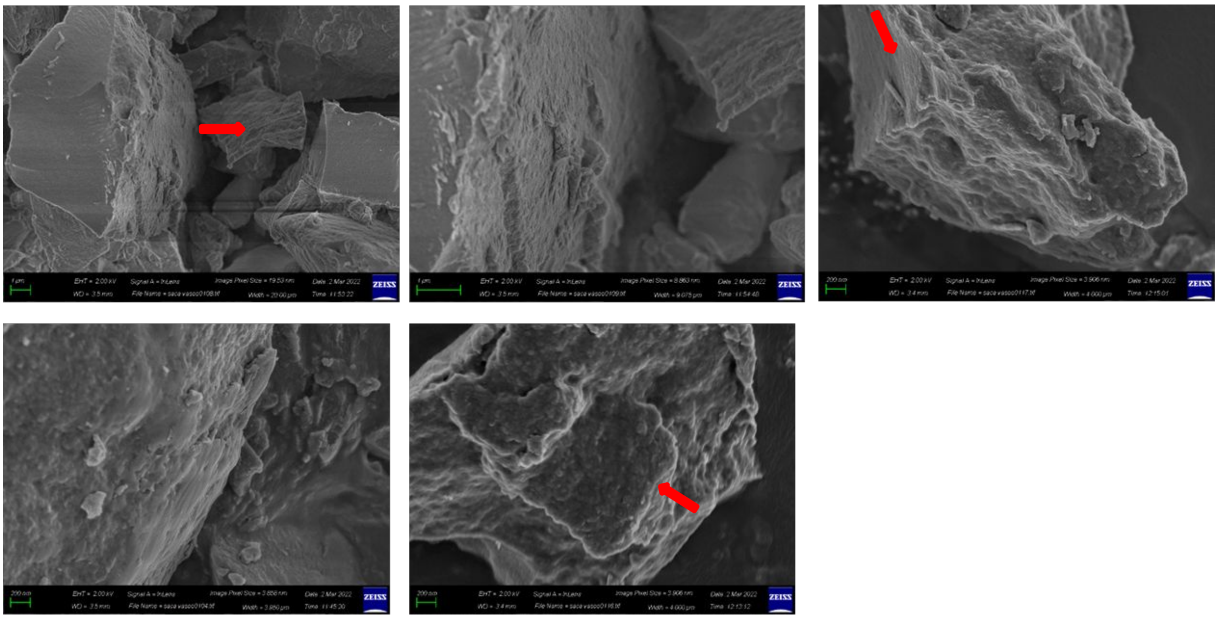

Figure S1. SEM images of Am(1) -SCA

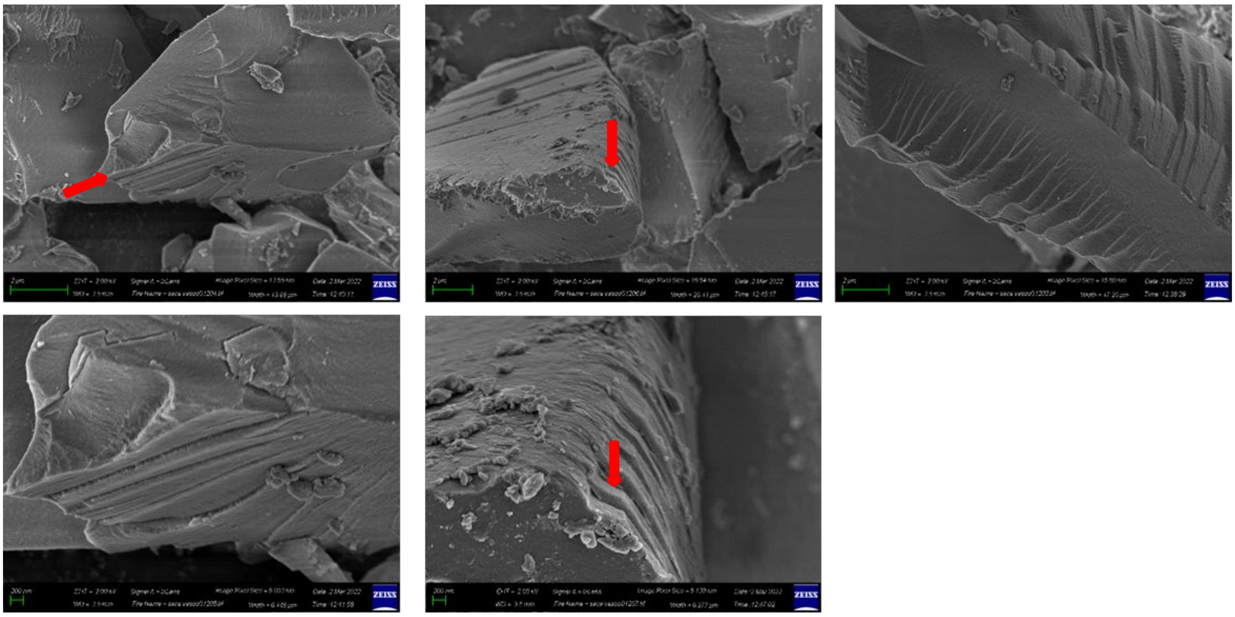

Figure S2. SEM images of Am(2) -SCA

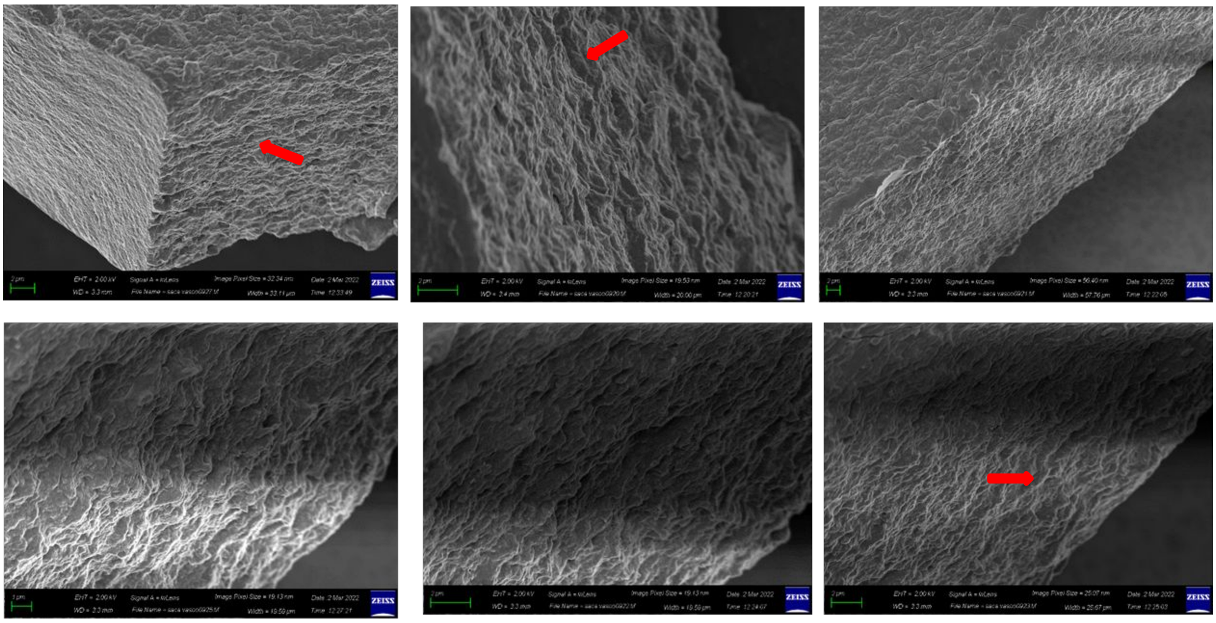

Figure S3. SEM images of Am(3) -SCA

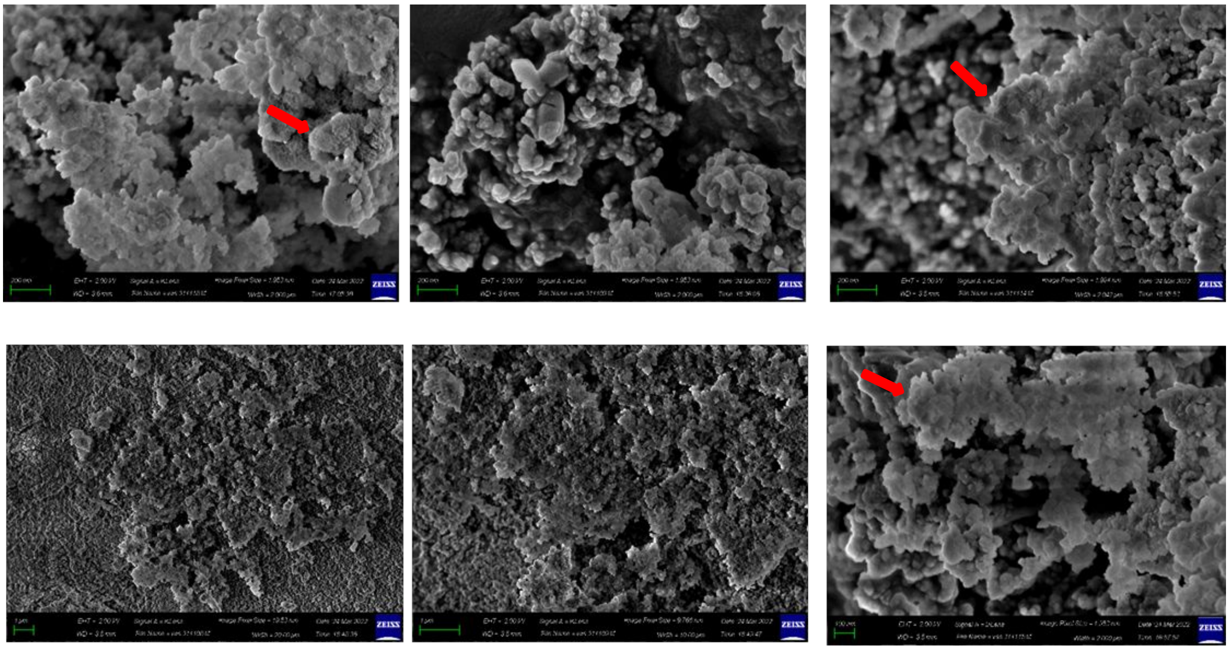

Figure S4. SEM images of Al -SCA

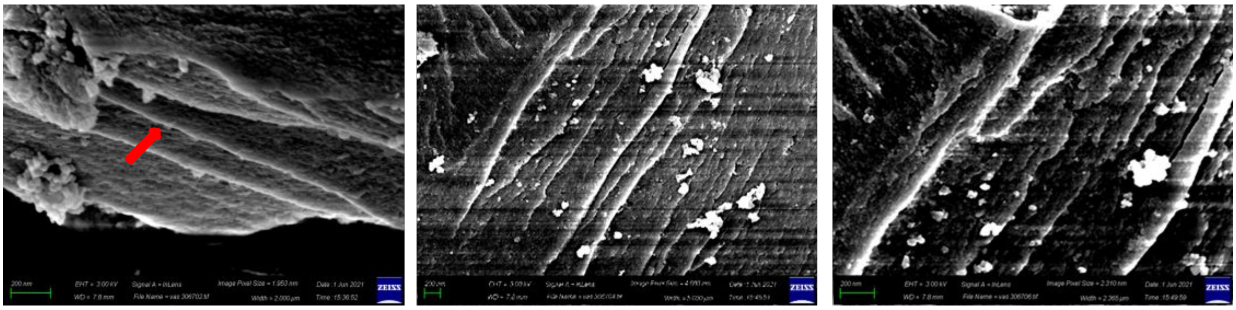

Figure S5. SEM images of Ar-SCA

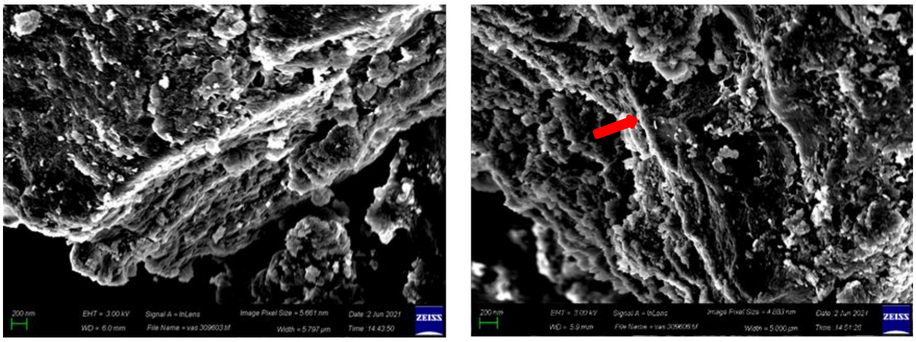

Figure S6. SEM images of Ch -SCA

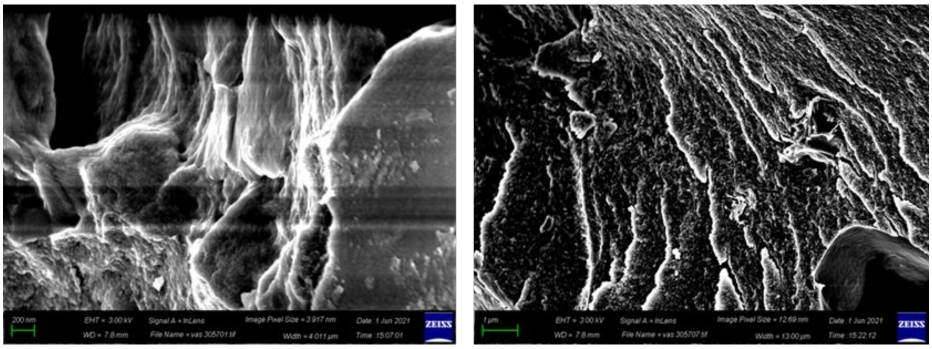

Figure S7. SEM images of Ep -SCA

2. DTA/TGA measurements

A **B**

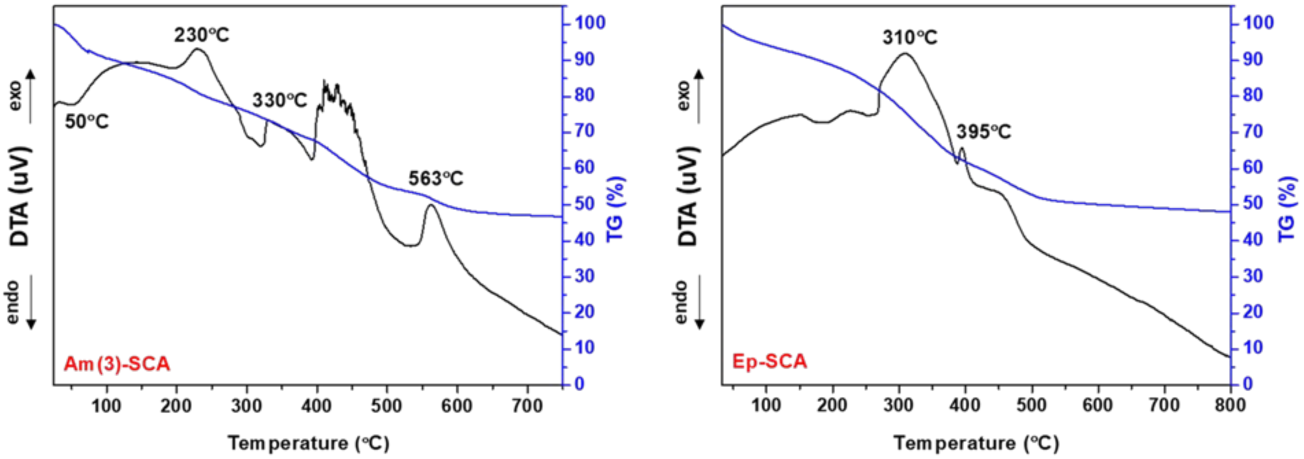

C

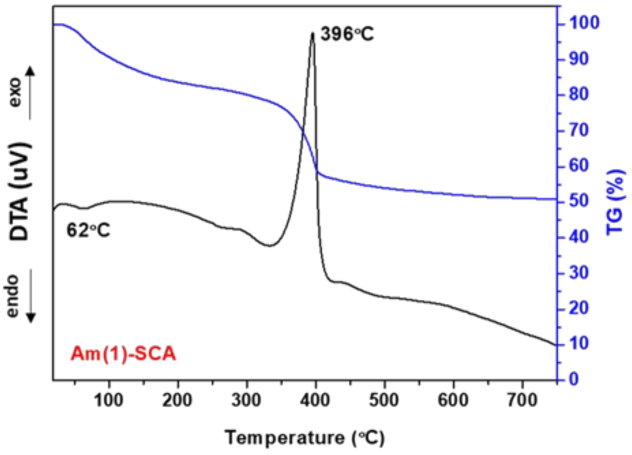
396°C
62°C
Am(1)-SCA
DTA (uV)
exo
endo
TG (%)
Temperature (°C)

D

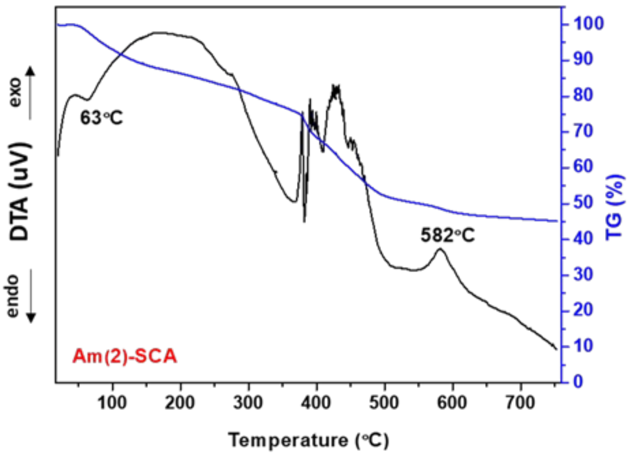
63°C
582°C
Am(2)-SCA
DTA (uV)
exo
endo
TG (%)
Temperature (°C)

E

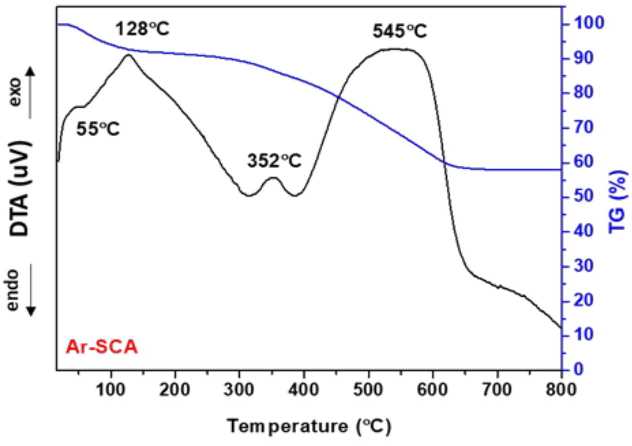
128°C
545°C
55°C
352°C
Ar-SCA
DTA (uV)
exo
endo
TG (%)
Temperature (°C)

F

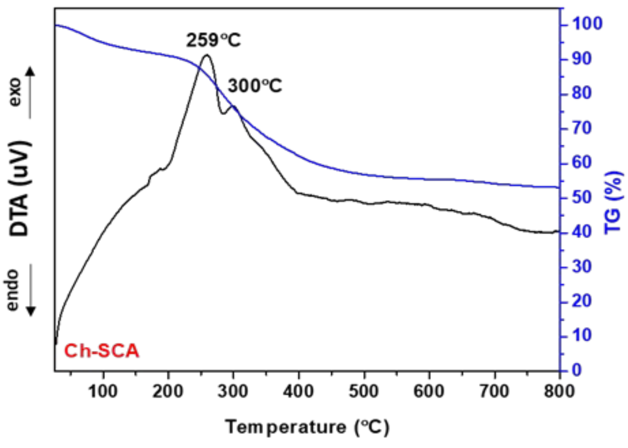
259°C
300°C
Ch-SCA
DTA (uV)
exo
endo
TG (%)
Temperature (°C)

G

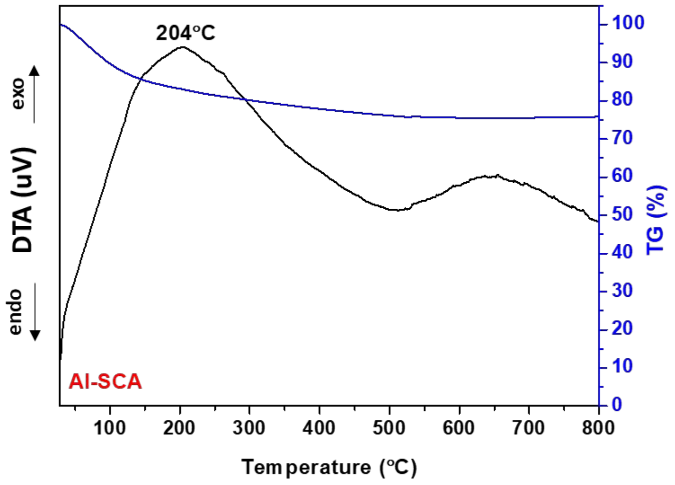

Figure S8. DTA and TGA curves of synthetic clay analogue with different silanes precursor (A. Am(1)-SCA, B. Am(2)-SCA, C. Am(3)-SCA, D. Ep-SCA, E. Ar-SCA, F. Ch-SCA and G. Al-SCA)

The thermal decomposition behavior of the synthetic clay analogues samples is analyzed using DTA/TGA technique. For the Am(1)-SCA an endothermic peak is initially observed at 62 °C (approximately 15%), which is assigned to the loss of physisorbed water from the surface, the edges, and in the interlayer spaces. This is followed by the weight loss (approximately 10%) occurring from 113 °C to 330 °C, which is attributed to the removal of organic groups. The third stage of weight loss (approximately 20%) observed an intense exothermic peak at 396 °C is corresponded to the destruction of the structure of the synthetic aminoclay analogue and the dehydroxylation. At temperatures exceeding ~400 °C, approximately 51% of the total mass was retained, primarily attributed to the presence of residual oxides, including silica and magnesium oxides.

Similar, thermal decomposition behavior is observed in Am(2)-SCA, Am(3)-SCA, Ep-SCA and Ar-SCA, respectively. The samples have three steps, where the first weight loss (Am(2)-SCA about 10%, Am(3)-SCA about 8%, Ep-SCA about 16% and Ar-SCA about 6%) occurs at (Am(2)-SCA: 63 °C, Am(3)-SCA: 50 °C, Ep-SCA: 185

°C and Ar-SCA: 55 °C) which is caused by adsorbed water loss. The second weight loss (Am(2)-SCA about 15%, Am(3)-SCA about 25%, Ep-SCA about 21 % and Ar-SCA about 5%) occurring from (Am(2)-SCA: 91 to 368 °C, Am(3)-SCA: 83 to 320 °C, Ep-SCA: 222 to 387 °C and Ar-SCA: 97 to 314 °C) is attributed to the removal of organic groups. While the third thermal event, at higher temperatures were observed various exothermic peaks with a weight loss of (Am(2)-SCA about 27%, Am(3)-SCA about 19%, Ep-SCA about 13% and Ar-SCA about 31%) is attributed to the decomposition of the structure of the synthetic clay analogues and the dehydroxylation. Finally, at temperatures exceeding ~600 °C, the Am(2)-SCA samples retained approximately 45% of the total mass, Am(3)-SCA about 47%, and Ar-SCA about 58%, respectively. In contrast, for the Ep-SCA sample, starting from ~500 °C and beyond, there was a residual total mass of around 48%, attributed to the presence of residual oxides, namely silica and magnesium oxides.

Finally, the samples Ch-SCA and Al-CA, the weight loss (Ch-SCA about 7% and Al-SCA about 12%) is due to the adsorbed water loss. Increasing the temperature, the main mass loss (Ch-SCA about 35% and –Al-SCA about 13%) observed exothermic peaks which are attributed to the removal of organic groups. At temperatures exceeding ~500 °C and above, the Ch-SCA sample retained approximately 53% of the total mass, attributed to the presence of remaining oxides (silica and magnesium oxides). In contrast, the Al-SCA sample exhibited thermal stability up to 800 °C, demonstrating no mass loss, and the percentage of remaining mass was approximately 76%, corresponding to the remaining oxides (silicon and magnesium oxides).

Table S1. Percentages of absorbed water and organic matter in organophyllosilicate clays.

Clay	Absorbed water (%)	Organic matter (%)
Am-SCA(1)	15	10
Am-SCA(2)	10	15
Am-SCA(3)	8	25
Ep-SCA	16	21
Ar-SCA	6	5
Ch-SCA	35	7
Al-CA	13	12

The antibacterial-drop test was performed according to our previous works[1, 2] with minor modifications which are explained. Aliquots of 25 µL of *E.coli* suspension from a fresh culture containing 2 x 10^7 CFU mL^{-1} were applied as a standing droplet on the surface of the Si-wafers (1 cm x 1 cm). Two control samples were also prepared, *E.coli* suspension without any substrate and *E.coli* suspension on the surface of untreated Si-wafers, and incubated at 37 °C for 16-18 h. After the interaction time, the samples were properly diluted with NaCl solution, spread uniformly in agar plates and the number of viable colonies that formed was counted. To confirm reproducibility, each sample was prepared and tested in triplicate.

The Langmuir–Blodgett (LB) technique is a precise method for preparing monolayer and multilayer thin films. It is a bottom-up approach that enables the precise control of

the layer thickness while allowing homogeneous deposition over large areas[3]. A KSV 2000 Nima Technology Langmuir−Blodgett device was used to prepare and deposit synthetic clay films at 21 ± 0.5 °C on Si wafers.

To further ascertain the bactericidal activity of the Am-SCA materials, Am-SCA(1) - Am-SCA (3) were deposited on the surface of Si-wafers using the Langmuir-Blodgett (LB) technique as described by Gournis et.al[4] in a similar work. For this purpose, an antibacterial drop-test was carried out using *E.coli* cells as a case of study at a final concentration of approximately 5 x 10^5 colony-forming units (CFUs) cm^{-2}. The percent of cell viability was determined by measuring the number of remaining viable cells after overnight incubation with the Si-wafer modified surfaces. Table S1 summarizes *E.coli* viability after interaction with the different samples. Blank Si-wafer was also tested as reference and insignificant effect on the viability of the cells was detected. As also shown above, Am-SCA materials exhibited significant decrease on the bacterial population which was more than 90%. The three amino clay analogues did not present substantial difference on their bactericidal activity hypothesizing that the -NH_2 terminal group possesses the pivotal role. To conclude, it is evident that these materials have strong antibacterial activities that pave the way for coating applications.

Table S2. Cell viability (%) of *E.coli* population after exposure for 16 h on the surface of Si-wafers modified with amino clay analogues. Means with different lowercase letters are significantly different ($p < 0.05$) according to Tukey's test.

	**Cell Viability (%)**
E.coli	**5 x 10^5 CFU cm^{-2}**

Blank Si-wafer	98 ± 2[b]
Am-SCA(1)_Si-wafer	5 ± 2[a]
Am-SCA(2)_Si-wafer	7 ± 3[a]
Am-SCA(3)_Si-wafer	8 ± 3[a]

References

[1] A. Kouloumpis, A.V. Chatzikonstantinou, N. Chalmpes, T. Giousis, G. Potsi, P. Katapodis, H. Stamatis, D. Gournis, P. Rudolf, Germanane Monolayer Films as Antibacterial Coatings, ACS Applied Nano Materials 4(3) (2021) 2333-2338.DOI: 10.1021/acsanm.0c03149.

[2] R. Fotiadou, I. Fragkaki, K. Pettas, H. Stamatis, Valorization of Olive Pomace Using Ultrasound-Assisted Extraction for Application in Active Packaging Films, International Journal of Molecular Sciences 25(12) (2024) 6541.

[3] R.Y. Gengler, A. Veligura, A. Enotiadis, E.K. Diamanti, D. Gournis, C. Józsa, B.J. van Wees, P. Rudolf, Large-yield preparation of high-electronic-quality graphene by a Langmuir-Schaefer approach, Small 6(1) (2010) 35-9.DOI: 10.1002/smll.200901120.

[4] N. Chalmpes, A. Kouloumpis, P. Zygouri, N. Karouta, K. Spyrou, P. Stathi, T. Tsoufis, V. Georgakilas, D. Gournis, P. Rudolf, Layer-by-Layer Assembly of Clay–Carbon Nanotube Hybrid Superstructures, ACS Omega 4(19) (2019) 18100-18107.DOI: 10.1021/acsomega.9b01970.
